# Amiodarone-Induced Myxedema Coma in Elderly Patients: A Systematic Review of Case Reports

**DOI:** 10.7759/cureus.40893

**Published:** 2023-06-24

**Authors:** Mohammad M Alnaeem, Khaled H Suleiman, Nadeen H Mansour, Bayan S Alwahsh, Abdulqadir J Nashwan

**Affiliations:** 1 School of Nursing, Al-Zaytoonah University of Jordan, Amman, JOR; 2 Department of Nursing, Hamad Medical Corporation, Doha, QAT

**Keywords:** systematic review, myxedema coma, cardiology, elderly, amiodarone

## Abstract

This systematic review aimed to explore whether elderly patients administered amiodarone were susceptible to developing myxedema coma. Utilizing the Cochrane guidelines, a comprehensive review of databases such as Medline (PubMed), Science Direct, CINAHL Cochrane, and Google Scholar was undertaken to examine case reports on amiodarone-induced myxedema coma. Following stringent criteria for inclusion, 12 pertinent case reports were identified. These findings suggested a high probability of myxedema coma development in patients who had been administered amiodarone. Specifically, patients who received an oral dosage of 100-200 mg of amiodarone were reported to have developed bradycardia and hypothermia alongside elevated thyroid-stimulating hormone (TSH) levels. Upon diagnosis, the majority of patients were treated with a regimen of levothyroxine and hydrocortisone medication. Despite the myriad potential causes of myxedema coma complicating the diagnosis, it was found that through a combination of clinical symptoms and serum TSH measurements, a confirmed diagnosis could be reached. Furthermore, it was observed that amiodarone-induced myxedema coma responded favorably to treatment with levothyroxine and glucocorticoids.

## Introduction and background

Myxedema coma is a severe and rare manifestation of longstanding hypothyroidism marked by impaired homeostasis [1]. This life-threatening presentation of hypothyroidism is characterized by a diminished mental state, hypothermia, and a general slowing of organ systems [2,3]. Typically, myxedema coma is associated with the terminal phase of severe hypothyroidism, which, if poorly managed, can result in fatal outcomes [4]. Triggers such as illnesses, myocardial infarctions, exposure to cold temperatures, or surgical procedures can precipitate myxedema [5]. This condition, affecting 0.22 individuals per million annually, predominantly targets women over the age of 60 [6]. Up to a quarter of affected patients may undergo either focal or generalized seizures, with potential signs of hypothermia, electrocardiographic abnormalities, cardiomegaly, decreased cardiac output, and hypotension [7]. In certain instances, myxedema can cause respiratory depression and gastrointestinal issues like anorexia, abdominal pain, constipation, fecal retention, and paralytic ileus [3].

Diagnosing myxedema can be challenging due to its presentation, which heavily relies on signs and symptoms. A common misconception is that patients may only be diagnosed with myxedema coma once they are comatose; however, the absence of edema or coma in most patients complicates this perception. If a patient's history and physical examination are consistent with hypothyroidism, combined with signs of stupor, disorientation, or coma, particularly in hypothermia, the diagnosis of myxedema coma may be more straightforward [3]. Meanwhile, thyroid-stimulating hormone (TSH) and free T4 are the most commonly utilized laboratory tests for evaluating thyroid function. Serum T4 levels are typically low in myxedema coma, while serum TSH levels may vary, indicating central or primary hypothyroidism [8].

The side effects of certain medications can trigger the onset of myxedema coma. Patients with Schizophrenia receiving lithium carbonate need to have their thyroid function routinely monitored before and during therapy, as hypothyroidism could necessitate discontinuation and proper treatment [9]. Nivolumab, a monoclonal antibody used in treating various cancers, can also induce myxedema coma and should be discontinued in severe hypothyroidism, though it can be resumed once the condition is stabilized [10]. Notably, amiodarone, at doses exceeding 200 mg/day, may result in amiodarone-induced myxedema coma (AIM) or hypothyroidism three months to two years after initiation [11]. Instances of myxedema coma in patients under amiodarone therapy without a previous thyroid disease history have been documented [12].

Amiodarone, a class III antiarrhythmic medication highly effective for supraventricular and ventricular arrhythmias, provides additional benefits for patients with left ventricular systolic dysfunction [13]. However, its frequent usage could disrupt thyroid function and other organ systems due to its heavily iodinated composition, raising concerns about thyrotoxicity in patients with cardiovascular disease or other health conditions [14,15]. Thyroid dysfunction is reported in 15-20% of patients taking amiodarone [16]. While it is posited that AIM can cause hypothyroidism, many cases remain undiagnosed [17,18]. This systematic review aims to ascertain whether elderly patients receiving amiodarone are at increased risk of developing AIM.

This review is of paramount importance as myxedema coma is a severe, life-threatening condition resulting from untreated or poorly managed hypothyroidism. By understanding the relationship between amiodarone, a widely used medication, and the onset of myxedema coma, healthcare professionals can better recognize, prevent, and manage this critical condition. The review addresses the potential underdiagnosis of AIM, which is crucial for timely interventions to reduce morbidity and mortality. It further brings to light the necessity for vigilant monitoring and therapeutic modifications among the vulnerable population, especially elderly women, who are disproportionately affected. This analysis may also pave the way for the development of guidelines concerning amiodarone usage and thyroid function monitoring, ultimately contributing to improved patient care and outcomes.

This review is guided by the Population, Issue of Interest, Comparison, Outcome, and Timeframe (PICOT) framework. The research question addressed here is: Are elderly patients administered amiodarone at risk of developing amiodarone-induced myxedema coma?

A preprint of this manuscript is available on Research Square [19].

## Review

Methods

Data Sources and Search Strategy

The authors followed the Preferred Reporting Items for Systematic Reviews and Meta-Analyses (PRISMA) guidelines to report this study. A systematic review of Medline (PubMed), Science Direct, CINAHL Cochrane database, and Google Scholar was performed to investigate the risk of developing myxedema coma in patients given amiodarone. Myxedema coma, amiodarone, induced, and side effects were all search terms used in this review. A limit on either English language and year of publication between 2016 and 2022 was imposed. All possible keyword combinations were searched, and a manual literature search was conducted. As an example of a literature search, as the following search terms: (myxedema coma) AND (induced) AND (Amiodarone), (myxedema coma) AND (amiodarone) AND (side effect) OR (adverse effect), ((myxedema coma) AND (amiodarone)) AND (side effect), ((myxedema coma) AND (Amiodarone)).

Study Selection and Eligibility

The (PICOT) framework guided the inclusion criteria. The following case reports were considered for inclusion in the review: The population was elderly patients; the intervention is receiving amiodarone; the control is inapplicable for our study; and the outcome is myxedema coma caused by amiodarone. In addition, patients with mental illnesses were excluded. To ensure study eligibility, the authors independently screened the titles and abstracts returned by the searches. If there were any disagreements among researchers about the titles of any study, or the abstracts needed to provide more information, the full text (available and requested) was reviewed to determine if the paper met the inclusion criteria. The full texts (available and requested) of all publications that were determined to meet standards potentially were then examined to determine final inclusion. Any disagreements between reviewers were accepted through consensus or the addition of a third reviewer.

Data Extraction and Synthesis

This review was registered in the PROSPERO databases (CRD42023399719). All our findings are case reports only. Two reviewers reviewed each study, and disagreements about data abstraction were resolved through consensus or by a third reviewer. Based on data abstraction elements, data were summarized in numeric form.

Results

**Figure 1:**
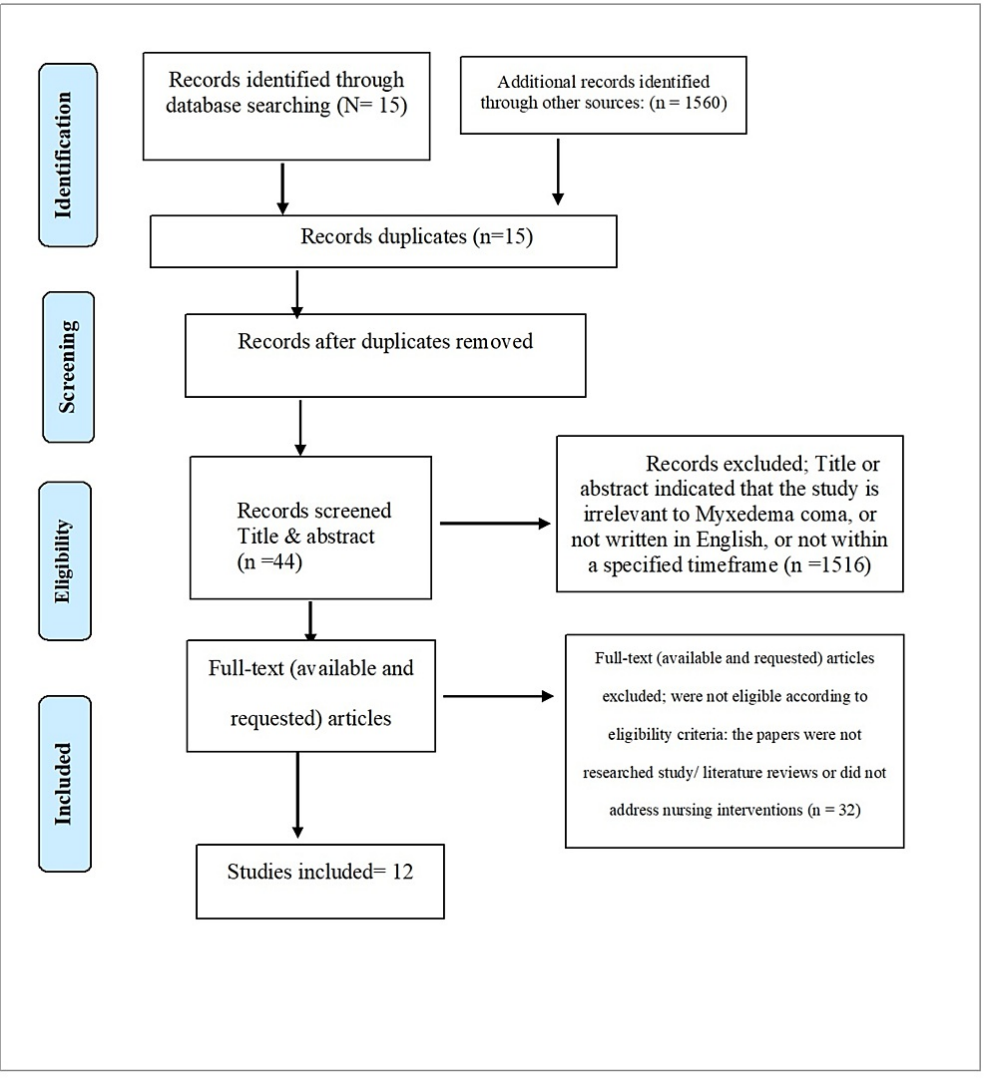
PRISMA flowchart PRISMA: Preferred Reporting Items for Systematic Reviews and Meta-Analyses

The study deletion process is shown in Figure 1. Our initial search yielded 1560 abstracts, of which 44 were found eligible based on initial screening and therefore underwent full-text review. Of the 44 case reports, 12 met the inclusion criteria and were included in the final evaluation.

By analyzing the study characteristic, most patients were female (N=8, 66.7%), and most patients’ age interval was 65-75 years old (41.6%). Also, the cases were reported in the USA, representing 75% of the case reports. Further, nine case reports (75%) of cases received care in the emergency department. Regarding the patient’s condition, most cases had atrial fibrillation; see Table 1.

**Table 1:** Characteristics of literature included in the review (N=12) ER: emergency room, ICU: intensive care unit

	N	%
Gender		
Female	8	66.7%
Male	4	33.3%
Age group		
65-75 years old	5	41.6%
76-86 years old	4	33.3%
87-97 years old	3	25%
Country		
USA	9	75%
SPAIN	1	8.3%
Portugal	2	16.6%
Department of care		
ER	9	75%
Internal Medicine Department	2	16.6%
ICU	1	8.3%
Years of Publishing		
2016-2018	4	33.3%
2019-2022	8	66.7%
Chronic medical history		
Atrial fibrillation & other illnesses	6	50%
Heart failure & Atrial fibrillation	3	25%
Atrial fibrillation only	1	8.3%
Other diseases only	2	16.6%

The current reviews used the Joanna Briggs Institute (JBI) critical appraisal tool for the included case reports. This tool consists of eight questions that include four possible choices (Yes, No, Unclear, not applicable), and each question was concise and clearly defined. The critical appraisal result for the case report shows a good quality of research evidence. One study scored (100%) for all questions [12], and four case reports scored 87.5% [2,20,21]. Also, five case reports scored 75% [11,22-25]. However, only one study scored 62.5% [26], as well only one study scored 50% [27]; see Table 2.

**Table 2:** JBI critical appraisal tool for case reports JBI: Joanna Briggs Institute

Authors (Year)	Q 1	Q 2	Q 3	Q 4	Q 5	Q 6	Q 7	Q 8	Percentage
Khdeir et al. (2020) [2]	Yes	Yes	Yes	Yes	Yes	Yes	No	Yes	87.5%
Kuroski et al. (2019) [11]	Yes	Yes	Yes	Unclear	Yes	Yes	No	Yes	75%
Martins et al. (2017) [12]	Yes	Yes	Yes	Yes	Yes	Yes	Yes	Yes	100%
Santos et al. (2017) [20]	Yes	Yes	Yes	Yes	Yes	Yes	No	Yes	87.5%
Rana & Ahmed (2019) [21]	Yes	Yes	Yes	Yes	Yes	Yes	Unclear	Yes	75%
Gonuguntla et al. (2020) [23]	Yes	Yes	Yes	Yes	Yes	Yes	No	Unclear	75%
Bui & Lazarus (2016) [24]	Yes	Yes	Yes	Yes	Yes	No	Unclear	Yes	75%
Villalba et al. (2019) [25]	Yes	Yes	Yes	Yes	unclear	Yes	No	Unclear	62.5%
Hawatmeh et al. (2018) [26]	Yes	Yes	Yes	No	No	No	No	Yes	50%
Kim & Syed (2020) [27]	Yes	Yes	Yes	Yes	Yes	Yes	No	Unclear	75%
Zagorski et al. (2020) [28]	Yes	Yes	Yes	Yes	Yes	Yes	No	Yes	87.5%
Armaghan et al. (2020) [29]	Yes	Yes	Yes	Yes	Yes	Yes	Unclear	Yes	87.5%

The abstracted data, including the study sample, setting, conclusion, and recommendation for all case reports, are presented in Table 3.

**Table 3:** Summary of the reviewed studies TSH: thyroid-stimulating hormone, CPK: creatine phosphokinase, MC: myxedema coma

Author name (year)	Design/sample	Settings	Conclusion	Recommendation
Khdeir et al. (2020) [2]	Case report (1)	Emergency Department	A few cases of people using amiodarone have been found to have a myxedema coma as a serious emergency.	Before administering amiodarone, it’s crucial to assess thyroid function.
Kuroski et al. (2019) [11]	Case report (1)	Emergency Department	In the literature, patients who experienced myxedema coma reported using amiodarone for three months and two years. However, this is the first case report of rapidly induced myxedema coma caused by amiodarone in a patient with an increased TSH.	When starting patients on amiodarone in the presence of an increased TSH, caution is required.
Martins et al. (2017) [12]	Case report (1)	Emergency Department	Depend on history and physical assessment findings such as bradycardia, hypotension, generalized edema, hypothermia, and hypoventilation with respiratory acidosis and supported by laboratory test of thyroid function (elevated TSH and low T4) help in early recognition of myxedema coma. Early start of adequate thyroid replacement therapy and corticosteroids, besides supportive measures, were necessary for the success of the treatment.	Focuses on future studies on treatment as the best thyroid hormone replacement therapy in myxedema coma patients. Patients under amiodarone therapy need close observation for any changes in thyroid function. Increase awareness about amiodarone-induced hypothyroidism to prevent myxedema coma that, considers a life-threatening condition.
Santos et al. (2017) [20]	Case report (1)	Emergency Department	Patients on amiodarone medication may get severe episodes of thyroid dysfunction.	Thyroid function monitoring in amiodarone-treated patients is significant.
Rana & Ahmed (2019) [21]	Case report (1)	Emergency Department	Myxedema coma manifested in this patient 12 months after initiating amiodarone.	NA
Gonuguntla et al. (2020) [23]	Case report (1)	Emergency Department	As early diagnosis and treatment would enhance outcomes, it is crucial to consider amiodarone-induced myxedema coma when making a differential diagnosis for patients who report altered mental status or hypothermia while using amiodarone.	When using amiodarone, patients should have their thyroid function tests thoroughly monitored for the first year.
Bui & Lazarus (2016) [24]	Case report (1)	Emergency Department	We present a case of hypothyroidism brought on by amiodarone that appeared as hypothermia.	NA
Villalba et al. (2019) [25]	Case report 3 females and one male patient	Department of Internal Medicine	Myxedema coma is an uncommon clinical condition associated with great mortality if not treated. All patients that present with bradycardia, bradypnea, hypoxemia, and elevated CPK should know thyroxin levels immediately to confirm the medical diagnosis and have baseline data before beginning treatment.	Hormone replacement therapy with T3 and T4 is preferred in a patient with past used amiodarone in treatment.
Hawatmeh et al. (2018) [26]	Case report (1)	Emergency Department	Our case reports further contribute to the literature and show that amiodarone significantly contributes to thyroid dysfunction, including hypothyroidism and myxedema coma.	While treating patients receiving amiodarone medication, medical professionals should be alert for thyroid dysfunction because early detection and management are crucial to achieving the best results.
Kim & Syed (2020) [27]	Case report (1)	ICU	Lethargy and disturbed mental status are nonspecific symptoms of myxedema coma that can occur without the more obvious skin abnormalities or myxedematous soft tissue alterations. There are no established treatment guidelines for myxedema coma. Despite the fact that the mainstays of therapy continue to be intravenous hydrocortisone and intravenous levothyroxine.	Conduct additional research on how T3 must be administered in accordance with the procedure for treating Myxedema coma that has not yet developed. It is crucial for doctors to consider myxedema coma as one of the differential diagnoses in patients on amiodarone who have an underlying thyroid condition. Polypharmacy should be considered while giving amiodarone to elderly patients with thyroid issues.
Zagorski et al. (2020) [28]	Case report (1)	Department of Internal Medicine	In this situation, we assume that amiodarone uses over a prolonged period caused his thyroid impairment.	It’s recommended that older persons taking amiodarone consider thyroid function testing. Healthcare providers should acknowledge myxedema coma as a potential diagnosis for amiodarone usage.
Armaghan et al. (2020) [29]	Case report (1)	Department of Emergency Medicine	Hypothyroidism brought on by a history of amiodarone medication can place a patient at a greater risk of developing MC.	It is significant to highlight that patient receiving long-term amiodarone medication should be continuously monitored for any indications of thyroid disease by thyroid panels.

By summarizing the included case reports, seven cases (58.3%) pointed out that 200 mg of amiodarone was prescribed [2,11,12,20,24,27-29], while four case reports (33.3%) did not mention the dose [21,22,26,30]; see Table 4.

**Table 4:** Findings of the review

	Number of studies	%	Reference
Dosage and route of Amiodarone			
200 Mg P.O	7	58.3%	[2,11,12,23,26-28]
100 Mg P.O	1	8.3%	[24]
Not reported	4	33.3%	[20,21,25,29]
Most common Symptoms *			
Altered mental status	10	34.5%	[12,20,21,23-29]
Bradycardia	8	27.6%	[2,11,12,20,25,27-29]
Hypotension	5	17.2%	[2,12,23,25,27]
Hypothermia	6	20.7%	[2,11,12,20,25,28]
Previous thyroid dysfunction			
Yes	3	25%	[11,25,27]
No	9	75%	[12,21,23,24,26,28,29]
Reporting (T3 & T4) Level			
Yes	6	50%	[2,21,23,24,27,28]
No	6	50%	[11,12,20,25,26,29]
Thyroid serum level (TSH)			
14-44	3	25%	[12,21,29]
50-100	4	33.3%	[11,20,25,28]
>100	4	33.3%	[2,23,24,27]
N/A	1	8.3%	[26]
Treatment in the hospital upon diagnosis			
Levothyroxine			
75 Mg I.V	1	8.3%	[27]
100 mcg I.V	4	33.3%	[11,12,23,28]
200 mcg I.V	1	8.3%	[20]
250 mcg I.V	1	8.3%	[29]
NA	5	41.6%	[2,21,24-26]
Hydrocortisone			
50 Mg I.V	2	16.6%	[28,29]
100 Mg I.V	3	25%	[11,12,27]
200 Mg I.V	1	8.3%	[20]
NA	6	50%	[2,21,23-26]

Altered mental status was also represented in 10 case reports (34.5%) [12,21,22,24-30], and eight cases (27.6%) reported bradycardia as a subsequent recurrent symptom among patients [2,11,12,21,26,28-30]. However, hypothermia and hypotension were reported about in half of the cases. Additionally, nine cases (75%) reported that the patients had no previous thyroid dysfunction [12,22,24,25,27,29,30], while three cases (25%) their patients had hypothyroidism [11,26,28]. Half of the included cases (50%) reported T3 and T4 levels [2,22,24,25,28,29], while the remaining did not [11,12,21,26,27,30].

For TSH, almost all cases reported the TSH level. Four cases (33.3%) pointed out TSH results between 50 and 100 mIU/L [11,21,26,29], the other four reported more than 100 mIU/L [2,24,25,28], and three (25%) reported TSH between 14 and 44 mIU/L [12,22,30]. Upon diagnosis with myxedema coma, treatment in hospitals includes levothyroxine and hydrocortisone. Different doses of levothyroxine were reported in seven cases; four cases (33.3%) pointed out 200 mcg, and three cases in each study had different doses, including 75mg [30], 100mg [28], and 250 mg [21]. However, five cases (41.6%) didn’t mention the dose of levothyroxine [2,22,25-27]. For hydrocortisone dose, seven cases mentioned the prescribed dose; three cases (25%) pointed out 100 mg of hydrocortisone [11,12,28], as well two cases (16.6%) pointed out 50 mg [29,30], and one study (8.3%) pointed out 200 mg of hydrocortisone [21]. On the other hand, five case reports did not mention the dose of hydrocortisone [2,22,24-27].

Discussion

Myxedema coma is a syndrome caused by a severe thyroid hormone deficiency. Amiodarone is an iodinated derivative of benzofuran that can cause hypo- or hyperfunction of the thyroid. On the other hand, myxedema caused by amiodarone therapy is extremely rare [31]. In the current review, the precipitating factor that led to the development of myxedema coma was a history of amiodarone therapy [2,12,21,22,28-30]. Amiodarone, which has a high iodine concentration, exerts its effects mostly through its structural relationship with thyroid hormones, and these two properties explain the changes in thyroid function reported. Amiodarone-induced thyrotoxicosis is more common in individuals who live in iodine-deficient areas and lead to several psychosocial impacts on adult patients [14,15,32,33]. While patients who consume enough iodine are more likely to develop hypothyroidism following amiodarone exposure, a condition known as AIM [34].

The incidence of AIM coma has been reported to range from 4% to 34% [35,36]. Female gender, older age, an underlying autoimmune thyroid disease, elevated baseline TSH levels, a starting dose of amiodarone greater than 200 mg/day, complex cyanotic heart disease, and residence in an iodine-sufficient region (e.g., the United States) are all risk factors for AIM [37-39]. Patients with myxedema coma in our review study were mostly female, older than 75 years, resided in an iodine-sufficient region (the United States), had atrial fibrillation, and started on amiodarone (200 mg/day).

The diagnosis of AIM depends on clinical manifestation (mental status, hypothermia, cold exposure, infection, drugs (diuretics, tranquilizers, sedatives, analgesics), trauma, stroke, heart failure, gastrointestinal bleeding), history or current amiodarone ingestion and the thyroid function tests [40,41]. Similarly, in the current review, most of the case reports showed the patients had elevated TSH, and 50% of the cases found that the patients had depressed T3 and T4 levels. Also, in three case reports in our review, the patients had a previous thyroid dysfunction (hypothyroidism). A mildly depressed thyroid panel with elevation in TSH was reported in the current review. It is critical to note that signs of thyroid dysfunction, as detected by thyroid panels, should be closely monitored in patients receiving chronic amiodarone therapy.

Patients with myxedema coma have common signs and symptoms such as hypothermia, bradycardia, hypotension, congestive heart failure, and hypoventilation with hypercapnia and respiratory acidosis [1,23]. Altered mental status, bradycardia, hypotension, and hypothermia are the most recurrent symptoms presented in our review for a patient diagnosed with myxedema coma who was on amiodarone, assessed by Glasgow Coma Scale and hemodynamic stability. The various possible causes of myxedema coma make diagnosis difficult. The symptoms of AIM are difficult to detect, especially in elderly patients with a history of heart disease who have further psychological symptoms in addition to inadequate adherence to their medication [15]. However, once diagnosed, often through clinical symptoms and serum TSH, this disease must be treated with thyroid hormone right away. Myxedema coma was successfully treated with levothyroxine and glucocorticoids, as presented in the included cases.

Myxedema coma is treated with hormone replacement therapy and supportive therapy. However, many countries still need to establish evidence-based treatment for myxedema coma because the disease is rare, and there is a lack of research in this area. Specific therapy entails giving levothyroxine [42,43], and most of the cases in the current review considered a maintenance dose of 100-200 g/day. Because absorption from the gut is unpredictable, the first dose should be administered intravenously, and this is consistent with all findings in this review that revealed levothyroxine was administered intravenously.

Myxedema treatment is associated with a risk of relative adrenal insufficiency; thus, glucocorticoid supplementation is needed [1,44]. In this review, five cases reported that glucocorticoid supplementation with hydrocortisone at 50-200 mg was administered intravenously. In addition, in this review, all cases reported that the patients received levothyroxine despite still taking amiodarone. This is supported by the current evidence suggesting that amiodarone can be continued in patients taking levothyroxine [41,45,46].

Moreover, it is critical to provide respiratory support through intubation, controlled mechanical ventilation, and supplemental oxygen therapy. Although the patient is hypothermic, external warming should be avoided because it can cause peripheral vasodilation and circulatory collapse. At room temperature, however, the patient can be covered with blankets [46].

A limitation of this systematic review, which primarily employs case reports to describe the relationship between amiodarone and the onset of myxedema coma, is the inherent weaknesses of case reports as a source of evidence. Case reports typically focus on an individual or a small number of cases and may not provide a comprehensive representation of the broader population. Additionally, they often lack control groups and blinding, making them susceptible to biases and confounding variables. The anecdotal nature of case reports means that causality cannot be definitively established, and the generalizability of findings is limited. Furthermore, the reliance on case reports may not sufficiently account for the diversity and complexity of clinical presentations and patient histories. It is important for future studies to employ more rigorous methodologies, such as randomized controlled trials or large-scale observational studies, to establish a more definitive and generalizable understanding of the association between amiodarone and myxedema coma.

## Conclusions

In conclusion, diagnosing myxedema coma is an intricate process due to its diverse causes and often subtle symptoms, which is further complicated in elderly patients with a history of cardiac conditions. Early detection is critical and usually involves a combination of clinical assessments and TSH measurements. Once diagnosed, immediate initiation of thyroid hormone therapy, particularly a combination of levothyroxine and glucocorticoids, is essential. The case studies analyzed in this review suggest effectiveness in treating myxedema coma with this therapeutic approach. However, it is crucial to note that the evidence is drawn from a limited source - case reports, which lack the generalizability and rigor of larger-scale studies. This underscores the need for ongoing outpatient monitoring, particularly in assessing the long-term effects of AIM and monitoring patient recovery. Therefore, while the case studies provide valuable insights, further research employing more robust methodologies is necessary for a comprehensive understanding. Rapid identification and management of myxedema coma are vital to reducing the risk of life-threatening complications and improving patient outcomes. Through enhanced awareness and vigilant patient monitoring, especially in high-risk populations, the healthcare community can effectively address the challenges of myxedema coma and contribute to improved patient care.
